# Aortic valve neocuspidization for aortic regurgitation associated with ventricular septal defect

**DOI:** 10.1093/icvts/ivab239

**Published:** 2021-09-09

**Authors:** Sivakumar Sivalingam, Maruti Haranal, Iqbal Hussain Pathan

**Affiliations:** Department of Cardiothoracic Surgery, National Heart Institute, Kuala Lumpur, Malaysia; Department of Cardiothoracic Surgery, National Heart Institute, Kuala Lumpur, Malaysia; Department of Cardiothoracic Surgery, National Heart Institute, Kuala Lumpur, Malaysia

**Keywords:** Aortic valve repair, Single-leaflet neocuspidization

## Abstract

**OBJECTIVES:**

Different methods of aortic valve repair have been described in the literature for aortic regurgitation (AR) associated with doubly committed subarterial ventricular septal defects. Our goal was to present our experience with aortic valve reconstruction of a single leaflet using the aortic valve neocuspidization technique in this subset of patients.

**METHODS:**

It is a retrospective review of 7 patients with doubly committed subarterial ventricular septal defects with significant (>moderate) AR who underwent the single-leaflet neocuspidization technique of aortic valve reconstruction from January 2016 to January 2019. Data were collected from medical records. All patients had thorough 2-dimensional echocardiographic assessment preoperatively and during the follow-up period. Primary end points were freedom from postoperative AR and freedom from reoperation and all-cause mortality within the follow-up period with secondary end points of freedom from thromboembolism and infective endocarditis.

**RESULTS:**

Out of 7 patients, 6 were male and 1 was female. There were no perioperative deaths. The mean follow-up period was 2.6 ± 0.8 years. No deaths occurred during the follow-up period. At the latest follow-up examination, only 2 patients showed mild AR and were asymptomatic. There was no documented event of infective endocarditis or thromboembolism during the follow-up period.

**CONCLUSIONS:**

The aortic leaflet neocuspidization procedure for the aortic valve is a relatively new concept. Availability of a template makes it an easily reproducible valve repair in paediatric patients with a single-leaflet abnormality. This technique preserves the remaining 2 normal leaflets, thus promoting the growth potential while maintaining near normal aortic root complex dynamics.

**Figure:**
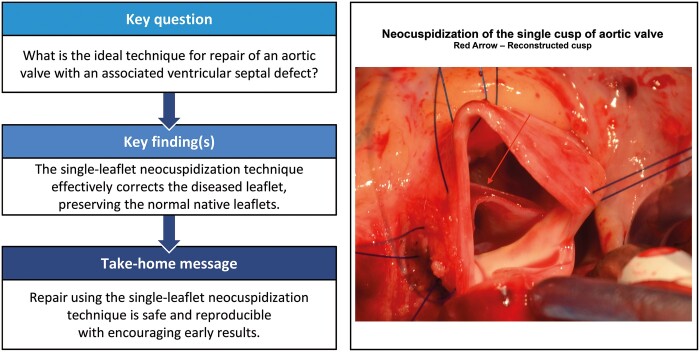

## INTRODUCTION

**Figure 1:**
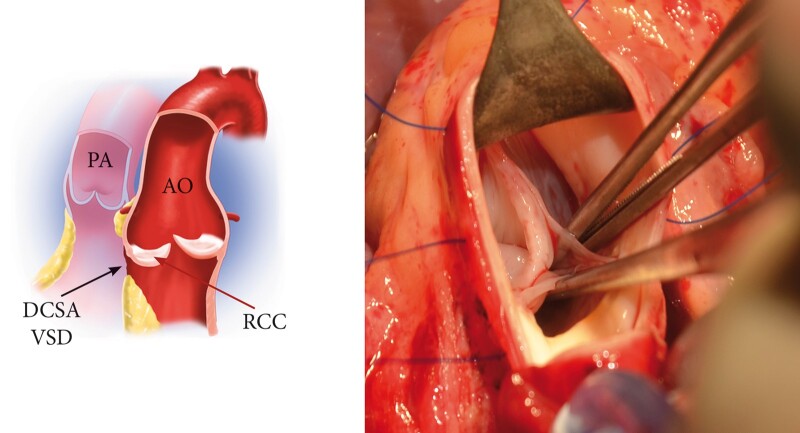
VSD with prolapse of an RCC. Ao: aorta; black arrow: DCSA VSD: doubly committed subarterial ventricular septal defect; maroon arrow: PA: pulmonary artery; RCC: prolapse of right coronary cusp.

Aortic regurgitation (AR) in the paediatric population results mostly from balloon valvuloplasty or aortic leaflet prolapse secondary to a ventricular septal defect (VSD), especially in a doubly committed subarterial (DCSA) (Fig. 1) and less commonly from a perimembranous type. Laubry and Pezzi [1] were the first to describe the syndrome of AR secondary to leaflet prolapse across the VSD. The reported incidence of aortic valve prolapse is 75% with subarterial VSD with a progression to echocardiographic AR of 78% [2]. The mechanisms proposed for an aortic valve leaflet prolapse leading to AR are the absence of muscular support with the resultant prolapse of the right coronary leaflet (non-coronary less often) of the aortic valve or the Venturi effect due to the close association of VSD with the prolapsing leaflet resulting in deformation of the aortic leaflet and progression to full-blown AR if not treated in a timely manner [3]. These patients present with variable degrees of AR due to single-leaflet deformity. In developed countries, due to early intervention for DCSA VSD, the aortic valve remains intact compared to the situation in developing countries, where there is a higher incidence of DCSA VSD presenting late with AR. The paediatric patients presenting with significant AR (>moderate) with VSD require surgical intervention to address the aortic valve with simultaneous closure of VSD.

Different surgical techniques for aortic valve repair have been described for the AR associated with this subset of patients with VSD. The aortic valve neocuspidization (AVNeo) technique was first described by Ozaki *et al.* [4] for trileaflet reconstruction of the aortic valve.

We report our experience of single aortic leaflet reconstruction using the AVNeo technique while preserving the remaining 2 native leaflets in this subset of patients.

## METHODS

We present a retrospective review of 7 patients who underwent simultaneous closure of a DCSA VSD and aortic valve repair using the AVNeo technique from January 2016 to January 2019; they were operated on by single surgeon at the National Heart Institute, Kuala Lumpur, Malaysia. During this period, 902 patients underwent closure of a VSD; 23 (2.5%) patients had associated AR. Of the 12 (52%) patients who had concomitant intervention on the aortic valve, 10 had aortic valve repair and 2 patients had aortic valve replacement. In addition to the 7 patients who had the repair using the AVNeo technique, 3 other patients with more than 1 cusp involved underwent repair using combined leaflet plication and commissural suspension techniques.

The institution’s ethics committee approved the study. The data were obtained from medical records. All patients underwent a thorough evaluation of their medical history and a clinical examination. All patients were examined with 2-dimensional echocardiography to assess the morphology of the aortic valve and the severity of the AR, the location and size of the VSD, any other intracardiac lesions and ventricular function. AR severity was graded using semi-quantitative methods. The patients with significant AR (>moderate grade) were considered for aortic valve repair along with VSD closure.

### Surgery

All patients were approached through a standard median sternotomy. Cardiopulmonary bypass was established with the aorta, bicaval cannulation and the left heart venting through the right superior pulmonary vein.

**Figure 2:**
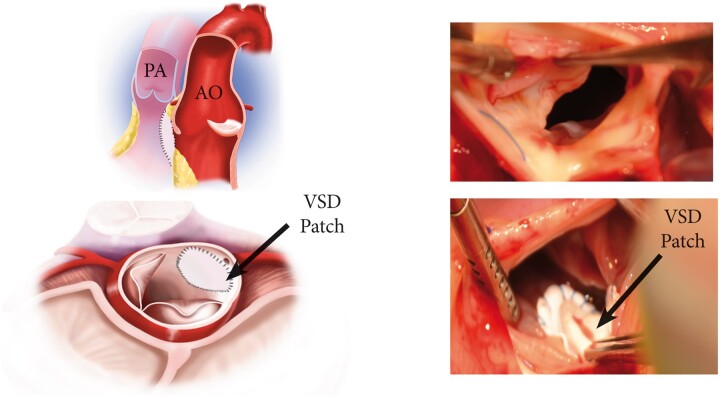
Closure of a VSD and excision of a right coronary cusp. Ao: aorta; black arrow: VSD: ventricular septal defect patch; PA: pulmonary artery.

Myocardial protection was done by moderate hypothermia (28–30° C) during cardiopulmonary bypass and following the cross-clamp using antegrade cold blood cardioplegia, repeated every 30 min and supplemented by topical cooling. A transpulmonary approach was used for VSD closure; the VSD was closed with a 0.4-mm polytetrafluoroethylene patch (Fig. 2).

**Figure 3:**
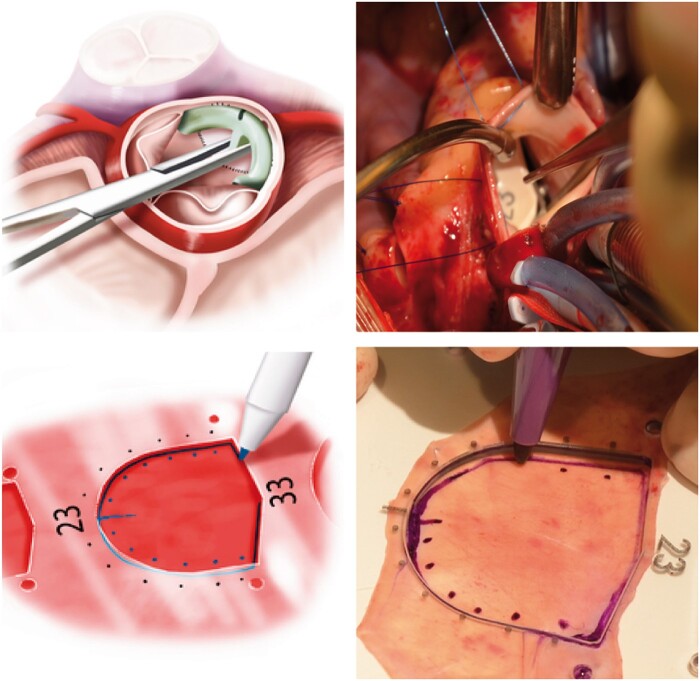
Measurement of cusp size using the sizer and the template used to create a new leaflet. Ao: aorta; PA: pulmonary artery.

**Figure 4:**
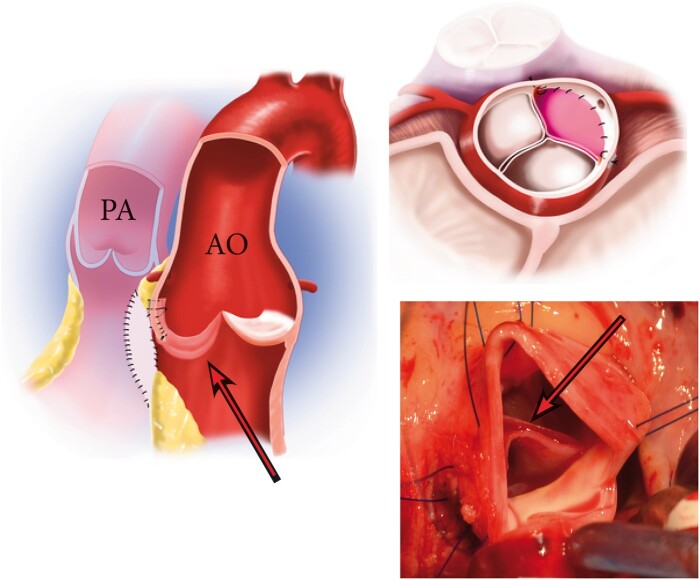
Aortic neocuspidization. Ao: aorta; PA: pulmonary artery; maroon arrow: reconstructed cusp.

A transverse aortotomy was performed 15 mm above the origin of the right coronary artery. The diseased coronary cusp (right coronary) was excised as completely as possible. Using the sizer provided for the AVNeo reconstruction, the appropriate cusp size was measured based on the circumferential length between the commissures at the level of the commissures (Fig. 3). A new leaflet corresponding to that measured by the sizer was trimmed from the pericardium placed on the template. Either a treated autologous pericardium (0.6% glutaraldehyde for 10 min) or tissue-engineered bovine pericardium (Cardiocel, Admedus, Perth, Australia) was used for the replacement of the single leaflet as described by Ozaki *et al.* [4] The annular margin of the pericardial leaflet was sutured with a monofilament 4/0 suture in a continuous manner (Fig. 4). Commissural coaptation was secured with an additional monofilament 4/0 suture. Additional procedures were performed as needed.

All patients were prescribed aspirin (5 mg/kg) for 3 months when they were discharged.

All patients were examined with follow-up 2-dimensional echocardiography to assess the status of the aortic valve repair.

Primary end points were freedom from postoperative AR, freedom from reoperation and all-cause mortality within the follow-up period with secondary end points of freedom from thromboembolism and infective endocarditis.

### Statistical analyses

Statistical analyses were performed using SPSS version 25.0 (SPSS, Inc., Chicago, IL, USA). Continuous variables are presented as mean ± SD. Categorical variables are presented as frequencies (%).

## RESULTS

### General

A total of 7 patients underwent simultaneous closure of VSD and repair of the aortic valve using the AVNeo technique during the study period. The mean age at surgery was 15.38 ± 9.70 years. The mean weight at surgery was 36.07 ± 16.41 kg. Patient characteristics are summarized in Table 1.

**Table 1:** Preoperative demographics

Patient	Age (years)	Gender	NYHA class	VSD type	Associated lesions	Severity of AR	Cusp involved	Aortic Annulus (mm)
1	8	M	2	DCSA	None	Severe	RCC	20
2	35	M	3	DCSA	Ruptured sinus of Valsalva	Severe	RCC	28
3	20	M	2	DCSA	Previous device closure of VSD	Moderate	RCC	30
4	12	F	2	DCSA	None	Moderate	RCC	23
5	9	M	2	DCSA	None	Severe	RCC	21
6	12	M	2	DCSA	Subaortic stenosis	Moderate	RCC	19
7	10	M	1	DCSA	None	Severe	RCC	23

AR: aortic regurgitation; DCSA: doubly committed subarterial; F: female; M: male; mm: millimetres; NYHA: New York Heart Association; RCC: right coronary cusp; VSD: ventricular septal defect.

### Operative data

Operative data are summarized in Table 2. The mean cross-clamp time was 141.2 ± 51.7 min, and the mean bypass time was 186.7 ± 81.6 min. One patient with a previous VSD device closure had a residual subarterial VSD (3.5 mm) with severe AR secondary to the tethering of the right coronary cusp (RCC) to the device. The device was extracted followed by excision of the RCC and the AVNeo of the RCC and VSD closure. Another patient presented with severe AR secondary to a ruptured right sinus of Valsalva through the VSD into the main pulmonary artery along with RCC prolapse. The ruptured sinus of Valsalva was repaired with 2 layers using Prolene 6/0 suture material followed by closure of the VSD. We attempted to repair the RCC with a cusp extension using autologous pericardium. However, there was residual moderate AR following the initial repair; hence, we resected the RCC and performed the AVNeo reconstruction of the RCC using tissue-engineered bovine pericardium. One patient with associated tricuspid regurgitation had a De Vega annuloplasty. One patient had an associated subaortic ridge that was resected.

**Table 2:** Operative data

Patient	Material used	Cusp size (mm)	Additional procedure	Intraoperative TOE
1	Autologous pericardium	23	None	No AR
2	Tissue-engineered pericardium	35	Repair of ruptured sinus of Valsalva	Mild AR
3	Autologous pericardium	35	Removal of VSD device	Mild AR
4	Tissue-engineered pericardium	29	None	No AR
5	Autologous pericardium	23	Tricuspid valve repair	Mild AR
6	Autologous pericardium	19	Subaortic resection	No AR
7	Autologous pericardium	25	None	No AR

AR: aortic regurgitation; TOE: transoesophageal echocardiogram; VSD: ventricular septal defect.

### Postoperative course

There were no perioperative deaths. The overall postoperative course was unremarkable except for 1 patient who required cardioversion for atrial fibrillation and 1 who had junctional rhythm that reverted to sinus rhythm. The mean stay in the intensive care unit was 50.5 ± 5.6 h, and the mean hospital stay was 7.75 ± 1.25 days.

### Follow-up period

The mean follow-up period was 2.6 ± 0.8 years. No patients died during the follow-up period. Two patients had mild AR at the latest follow-up and were asymptomatic. There was no evidence of thromboembolism, infective endocarditis or reinterventions during the follow-up period.

## DISCUSSION

The DCSA VSD has a higher prevalence in the Asian population. The presentation of these patients is often delayed due to a physiologically insignificant shunt and the absence of symptoms. Therefore, most of these patients present with variable degrees of AR secondary to a deformed prolapsing leaflet. Tatsuno *et al.* [5] first described the natural history of this anomaly and reported good results with closure of the VSD alone when the AR is mild. Children presenting with AR due to the prolapse of a single cusp secondary to underlying subarterial VSD with 2 healthy cusps is a real challenge. Valve replacement is a less ideal option for the paediatric population, and the lack of the availability of a proper size match of the prosthesis is a major limitation. The Ross procedure is technically more complex in patients with failure to repair or early failure. Using different repair techniques with the potential to fail while the patient is on the operating table or early after surgery is always a concern.

In 1960, Starr *et al.* [6] presented his series of encouraging results from aortic valve repair in patients with DCSA VSD. In 1963, the Mayo Clinic published its experience with 30 patients in whom the aortic valve cusps were reconstructed and repaired [7]. In 1992, Bonhoeffer *et al.* [8] described a new two-patch technique wherein the problem of lack of support of the aortic cusp was eliminated by creating a new aortic annulus and giving it a support.

Yacoub *et al.* [9] suggested anatomical correction of the defect. According to them, the mechanism responsible for the prolapse is disconnection of the aortic annulus responsible for the continuity between the aortic sinus and the leaflet from the ventricular septum. This group advocated primary closure of the VSD using a series of pericardial pledgetted mattress sutures inserted into the right ventricular side of the crest of the interventricular septum through the aortic valve annulus and then through the thin portion of the prolapsed aortic sinus, resulting in direct closure of the VSD with simultaneous elevation of the aortic annulus, thus addressing both the dilated annulus and prolapsing leaflet. Despite acceptable results from the anatomical correction, there are concerns of undue tension resulting in a conduction defect or recurrence of VSD.

AR in association with VSD results from the deformed prolapsing leaflet, which leads to the origin of different repair techniques. A number of surgical valvuloplasty techniques have been described with varying results. The Trusler *et al.* [10] repair of plication is a widely adopted method to address the prolapsing aortic valve leaflet, but there are only a few reports of long-term outcomes [11]. In 1990, Carpentier *et al.* presented their results from the triangular resection of the prolapsed cusp, annuloplasty and reinforcement of the aortic wall followed by transaortic patch closure of the VSD [12]. Despite improvements in the surgical management of this subset of patients, there is still a lack of consensus as to the ideal technique to address this syndrome of AR because of variable results reported by different groups [13, 14].

Song *et al.* [15] published their experience with single-leaflet replacement with bovine pericardium in patients with VSD-AR. All of the operations were performed by a single surgeon. The lengths of the corresponding free edges of the other 2 leaflets were measured as far as the distance between the nodulus of Aranti and the commissure to determine the dimensions of the new leaflet. The study showed good midterm results; however, the long-term results are yet to be determined.

Professor Ozaki *et al.* [4] described a technique of AVNeo using glutaraldehyde-treated autologous pericardium, wherein templates are available to cut fixed pericardium as per measurements from the sizers. The major benefit of the AVNeo technique is the availability of a template for sizing the desired leaflet with virtually no reason for failure. Furthermore, this technique is able to achieve a larger coaptation zone and decrease the mechanical stress on the commissures and the annulus.

In DCSA VSD, the superior border of the VSD is uniformly adjacent to the hinge point of the RCC, and the VSD has a shallow scooped out or half-moon shape. Consequently, the jet of blood through the defect is maximally exposed to the RCC. The aortic insufficiency develops because of the lack of support for the aortic dilatation of the aortic cusp. The downwards and outward displacement of the annulus in the region of the RCC into the right ventricle leads to progressive sagging and enlargement of the body of the cusp, which results in failure of the coapting surface of the cusp to meet the other 2 cusps and in progressive elongation of the free margin in the RCC. Because the defect only affects the RCC, performing a neocuspidization procedure on the single RCC will effectively correct the regurgitant lesion.

The single-leaflet reconstruction of the RCC using the AVNeo technique corrects the aortic valve incompetence in the following ways:

1. Because the leaflet size is based on the inter-commissural distance, the reconstructed leaflet is able to correct the enlarged body of the cusp.
2. The reconstructed aortic valve has the coaptation point at the level of the commissures compared to the other 2 normal cusps, which anatomically are below the level of the commissures. However, the downwards displacement of the RCC, which lies at a lower level compared to the other 2 cusps, compensates for the higher position of the leaflet, which ultimately will lie at the same level as that of the other normal cusps.
3. In the paediatric population, reconstructing just 1 leaflet using a biological material and leaving the other 2 normal cusps intact allows the annulus to grow naturally. In addition, the reconstructed leaflet, which is adjusted to the enlarged and displaced annulus, helps to remodel the annular-cusp complex along with the growth of the annulus.

Hence the advantage of this reparative technique compared to those that involve repairing the native leaflet is that reconstructing the entire leaflet by adjusting the height and size according to the disproportionally enlarged annulus helps stabilize the repaired leaflet because it maintains a good coaptation height and at the same time aids in remodelling the deformed annulus. Besides, we believe the other 2 normal leaflets and annulus allow the natural growth of the cusp-annular complex, which will achieve long-term durability. This outcome is appropriate for children who are still growing. If the repair lasts until adulthood and if the need for reoperation arises, one could consider replacement of the aortic valve, which is feasible in the older patients.

The goal of reporting this study is to share our experience with this relatively new technique to address a single leaflet in paediatric patients with cusp prolapse secondary to DCSA VSD while preserving the other 2 native leaflets, giving the maximum benefit of the natural dynamics of the aortic valve complex mechanism and simultaneously providing the native leaflet a chance for growth. During the follow-up period, there was no recurrence of regurgitation or development of new-onset aortic stenosis. We prescribed aspirin for 3 months with no report of any thromboembolism.

Our patient population was different from the population in Ozaki’s original report, wherein 75% of the patient population had a small aortic annulus, which is also a concern when we deal with paediatric patients. The AVNeo is gaining more popularity due to template-guided creation of a new leaflet, thereby making the technique more standardized and reproducible. Furthermore, because the templates come with smaller sizers for the paediatric patients, it is possible to use this technique in the setting of the small aortic valve annulus found in this group of patients. The AVNeo procedure showed excellent results in the adult population; nevertheless, paediatric cases accounted for a small proportion and further follow-up is needed, particularly in the paediatric population.

### Limitation

It is a retrospective single-centre study with a limited number of cases and a short follow-up period.

## ABBREVIATIONS

AR: Aortic regurgitation
AVNeo: Aortic valve neocuspidization
DCSA: Doubly committed subarterial
RCC: Right coronary cusp
VSD: Ventricular septal defect
